# Deep Learning (nnU-Net)-Based Segmentation of Primary HPV-Positive OPSCC: Contrast-Enhanced T1-Weighted Fat-Suppressed Versus Non-Contrast-Enhanced T2-Weighted Fat-Suppressed MRI (Paired Single-Center Study)

**DOI:** 10.3390/diagnostics16050658

**Published:** 2026-02-25

**Authors:** Viktoriia Zarovniaeva, Ramkumar Rajabathar Babu Jai Shanker, Amogh Shetty, Daniel T. Ginat

**Affiliations:** 1 Department of Radiology, University of Chicago, Chicago, IL 60637, USA; kuobah12@gmail.com (V.Z.); rbramkumar@gmail.com (R.R.B.J.S.); 2 Rad-Lab.ai, Dover, DE 19904, USA; 3 Department of Biology, Rensselaer Polytechnic Institute, Troy, NY 12180, USA; shettyamogh11@gmail.com

**Keywords:** magnetic resonance imaging (MRI), convolutional neural networks (CNNs), oropharyngeal squamous cell carcinoma (OPSCC), non-contrast MRI (gadolinium-free), automatic tumor segmentation (auto-segmentation), segmentation performance, performance comparison, contrast vs. non-contrast

## Abstract

**Background/Objectives:** While deep learning-based AI algorithms have been shown to perform well for OPSCC tumor segmentation, the relative value of contrast-enhanced versus T2-weighted sequences for automated segmentation has not been systematically evaluated. In this study, we compared the sequence-specific deep learning performance on contrast-enhanced T1-weighted fat-suppressed and T2-weighted fat-suppressed MRI in HPV-positive OPSCC. **Methods:** Pretreatment MRI from 39 patients with paired sequences from a single center were retrospectively analyzed. OPSCC primary tumors were manually segmented using both sequences, which served as the ground truth. Three sequence-specific configurations were evaluated: contrast-enhanced (CE), T2-only, and combined CE + T2. Quantitative evaluation was carried out on aggregated out-of-fold predictions using Dice score (primary), Surface-Dice@2mm (secondary), and other boundary and volumetric metrics, and paired comparisons (combined vs. T2-only; CE-only vs. T2-only) were performed using an exact Wilcoxon signed-rank test. Qualitative evaluation was performed on 4-point ordinal acceptability ratings recorded using a blind reader study, and the ratings were compared using the exact Wilcoxon signed-rank test (pairwise) and dichotomized acceptability using the McNemar test. **Results:** Median Dice was comparable across configurations (0.63 for CE + T2, 0.60 for T2-only, and 0.55 for CE-only). Median Surface-Dice@2mm was highest for the combined configuration (0.62), followed by CE-only (0.6) and T2-only (0.57). Median ASSD were 2.71, 2.98, and 2.98 mm, and median HD95 were 11.39, 15.0, and 11.3 mm for combined, CE, and T2, respectively. The median GTV differences (−1.31, −1.29, and −1.49 mL for combined, T2, and CE, respectively) showed a slight bias toward under-segmentation across all configurations. No significant differences in Dice scores were observed for combined vs. T2 (*p* = 0.11) or contrast-enhanced vs. T2-only (*p* = 0.98). Similarly, qualitative analysis also showed no evidence of performance difference for ratings and acceptability rates across sequence configurations (paired Wilcoxon, *p* ≥ 0.35; McNemar, *p* = 1.00). **Conclusions:** In this single-center study, the segmentation performance using non-contrast sequences was comparable to that using both contrast-enhanced and non-contrast sequences. The drop in performance when the contrast-enhanced sequences were excluded from the combination was not significant. These findings justify multi-center validation to support the feasibility of contrast-sparing automated primary OPSCC segmentation when use of contrast agents is contraindicated.

## 1. Introduction

Oropharyngeal squamous cell carcinoma (OPSCC) is a rapidly growing malignant tumor of the head and neck. Over 450,000 cases of OPSCC have been registered worldwide [1]. Squamous cell carcinoma comprises approximately 90% of oropharyngeal neoplasms [2]. There are two etiological types of OPSCC: OPSCC caused by noxious agents and human papillomavirus (HPV)-associated carcinoma [3]. Although a multitude of etiological factors contribute to the pathogenesis of both oral squamous cell carcinoma (OSCC) and oropharyngeal squamous cell carcinoma (OPSCC), alcohol and tobacco consumption continue to be the most significant risk factors [4]. Other pathological conditions, such as the presence of oral precancerous lesions like leukoplakia, erythroplakia, and lichen planus, as well as infectious agents like HPV, hepatitis C virus (HCV), and Epstein–Barr virus (EBV), have been concurrently associated with additional risk factors [5]. HPV is responsible for approximately 25% to 35% of all cases of squamous cell carcinoma of the head and neck. Most diagnosed cases are malignancies of the tonsils and base of the tongue [1,6].

Accurate diagnosis and determination of tumor extent are key steps in selecting the treatment strategy for OPSCC. In this context, magnetic resonance imaging (MRI) plays an essential role in the diagnosis, staging, and follow-up of head and neck tumors. Precise identification and delineation of tumor margins are critical for radiotherapy treatment planning, where the goal is to target the tumor area and spare surrounding normal tissues [7]. Tumor delineation is especially challenging in the oropharyngeal region because of complex anatomy and the presence of various tissue types. Currently, in the clinical setting, tumor boundaries are outlined manually by a trained radiologist. However, manual segmentation of primary tumors is time-consuming and is subject to inter-observer variability [8]. Therefore, fully automated, highly precise, and accurate and consistent segmentation techniques are valuable to improve clinical workflow and patient outcomes.

Gadolinium-based contrast agents (GBCAs) give the opportunity to shorten T1 relaxation times and increase signal intensity on T1-weighted images. However, the use of contrast agents also carries the risk of side effects, including Nephrogenic Systemic Fibrosis (NSF) in patients with renal impairment and allergic reactions [9]. Non-contrast MRI T2-weighted images with fat suppression could be a potential alternative for the segmentation of tumors. Improving the ability to detect tumors and precisely outline margins on non-contrast/low-dose contrast imaging could be beneficial in cases where administering a contrast agent is not feasible due to cost or availability limitations in certain regions and contraindications. Automated segmentation of tumors on non-contrast MR sequences could provide streamlined workflows and ensure reproducibility across diverse healthcare environments.

In recent years, several algorithms have been proposed that either built on the U-net architecture or utilized novel architectures. The nn-UNet (no-new-UNet) proposed by Isense et al. [10] automated the hard tasks of finding the right configuration of the U-net network architecture, preprocessing, and training parameters. The Attention U-net proposed by Otkay et al. [11] introduced network features that help the algorithm focus on salient anatomical features and suppress irrelevant features. The UNETR and SwinUNETR architectures proposed by Hatamizadeh et al. [12,13] utilized the transformer architecture that has shown excellent capabilities in capturing local and global features as well as contextual information.

Recently, deep learning-based artificial intelligence algorithms, such as Convolutional Neural Networks (CNNs), have demonstrated strong performance in medical imaging tasks, including tumor detection, segmentation, and characterization [14]. The U-Net, which uses encoder–decoder architecture, has achieved high segmentation accuracy by capturing complex high-level semantic information and precisely localizing tumor regions [15]. In recent studies, CNNs have been demonstrated in the segmentation of various cancer types, with Dice coefficients ranging from 0.74 to 0.95 [16,17,18,19]. However, prior work on AI segmentation of oropharyngeal tumors has primarily focused on using multi-parametric (both contrast-enhanced and non-contrast) sequences for tumor segmentation [20,21,22]. Direct systematic comparisons of contrast-enhanced versus non-contrast sequences for automated primary tumor segmentation remain limited.

Our work aims to address these gaps by evaluating the sequence-specific performance of CNN algorithms in segmenting OPSCC primary tumors on contrast-enhanced and non-contrast MRI. Specifically, we (i) trained separate U-Net models on contrast-enhanced MRI, non-contrast MRI, and their combination, (ii) evaluated them with volumetric and boundary metrics, and (iii) compared their quantitative and qualitative performance on paired test scans.

## 2. Materials and Methods

### 2.1. Dataset

For this retrospective study, we used pre-treatment baseline MRI images from the OPTIMA II Phase 2 Open-Label Nonrandomized Controlled Trial [23]. This cohort included images from 62 HPV-positive (histologically proven) patients collected between October 2017 and February 2020. This trial was conducted under a HIPAA-compliant protocol approved by the MD Anderson Institutional Review Board (RCR03-0800), and informed consent was waived. The images were acquired using GE HealthCare (Discovery MR750w, Signa Explorer, Signa Architect, Optima MR450w, Chicago, IL, USA) or Philips Medical Systems (Achieva, Ingenia, Amsterdam, The Netherlands) systems at 1.5 and 3T, with slice thicknesses set at 3–4 mm. The protocol included 2D T2-weighted fast spin-echo with fat suppression (T2w) and 3D T1-weighted high-resolution isotropic volume excitation after contrast (Multihance or Dotarem) injection with fat suppression (T1gd). The details of scanner-specific parameters (e.g., TE/TR values, flip angle, bandwidth) for both CE-T1W-FS and T2W-FS sequences across GE and Philips systems are summarized in Supplementary Table S1.

**Figure 1:**
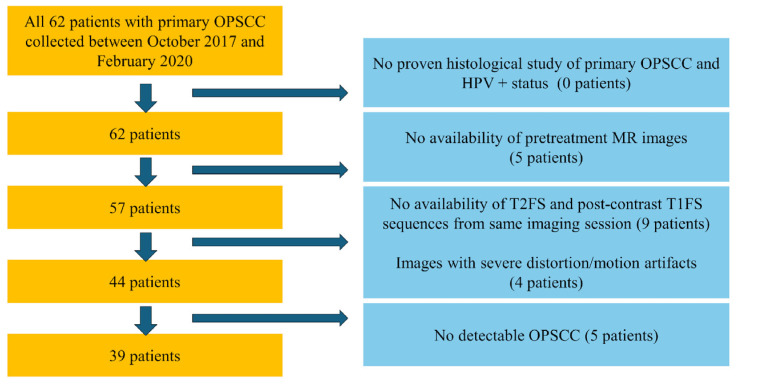
Patient selection process flowchart.

In this study, we only included patients who had both contrast-enhanced T1-weighted fat-suppressed and non-contrast T2-weighted fat-suppressed (T2W-FS) sequences acquired during the same imaging study (Figure 1). We excluded patients whose images missed either sequence (CE-T1W-FS or T2W-FS) or if their images contained artifacts or featured no visible primary OPSCC tumors.

### 2.2. Reference Standard (Tumor Annotations)

**Figure 2:**
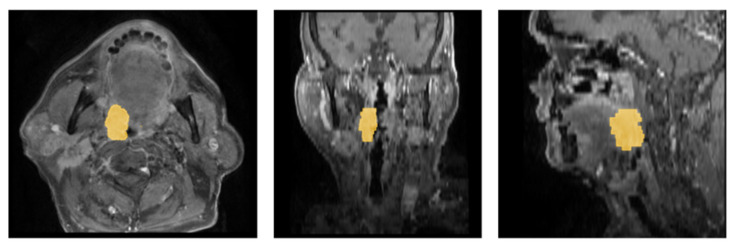
Illustration of tumor boundaries annotated using 3D slicer of a sample patient (IRB14-0749:16533607) on CE-T1W-FS showing the tumor (T3, N1, Mx) located at the right tonsil, right lateral oropharynx wall, with GTV of 11.31 mL.

For each patient, a radiologist with >10 years of clinical experience viewed both CE and T2 images side by side on 3D Slicer (v5.6.2) [24] and created the mask by outlining the tumor boundaries on the contrast-enhanced sequence (Figure 2). While the CE images served as the primary sequence for GTV delineation, T2 images were used as a secondary anatomic reference during side-by-side review and to support accurate boundary delineation. When the apparent tumor extent differed between CE and T2 sequences, enhancing tumor on CE images was used to define the tumor boundaries, and any additional T2 hyperintensities were included only if they were judged to represent solid tumor rather than edema. These segmentations were reviewed by a senior board-certified neuroradiologist (>17 years of experience). Discrepancies in outlined boundaries were resolved by consensus, and corrections proposed by the senior neuroradiologist were made during structured teleconference sessions. T2W-FS images were co-registered to CE-T1W-FS space using rigid/affine (SimpleITK v2.5.2) and SyN (ANTs v0.6.1) [25], and the resulting alignments were visually confirmed by the radiologist.

### 2.3. Data Splits/Experimental Design

**Figure 3:**
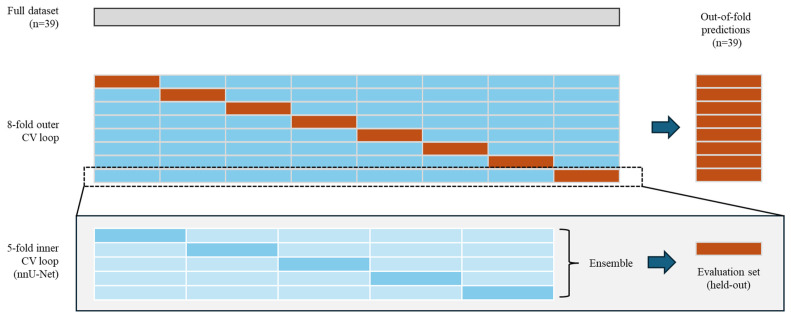
Data partition strategy and experimental design. The figure illustrates the per-configuration (CE-T1W-FS, T2W-FS, or CE-T1W-FS + T2W-FS) design and patient splits, which were fixed across all three configurations. The full cohort (*n* = 39) was evaluated using an **8-fold outer cross-validation** loop: **orange** indicates the held-out evaluation fold and **light blue** the remaining development set. Within each outer loop, a **5-fold inner CV** trained nnU-Net models and formed an ensemble (**dark blue** = inner validation folds; **light blue** = inner training folds).

Since the sample size was limited, we used a nested cross-validation (CV) design to train and evaluate the models (Figure 3). The nested CV design was chosen to ensure that the generalization capacity (more patients in the training set) was balanced with the ability to provide unbiased assessment of performance. In this design, the full dataset is divided into eight outer folds using a stratified sampling based on GTV. Within each outer fold, the training set (88%) is split into five inner folds using random sampling and U-Net models were trained on each inner fold. The predictions from the inner-fold models were ensembled (average) to predict on the held-out set (12%) for that outer fold. These out-of-fold (OOF) predictions were then aggregated from all outer folds to provide patient-level performance estimates that are unbiased and leakage proof.

**Figure 4:**
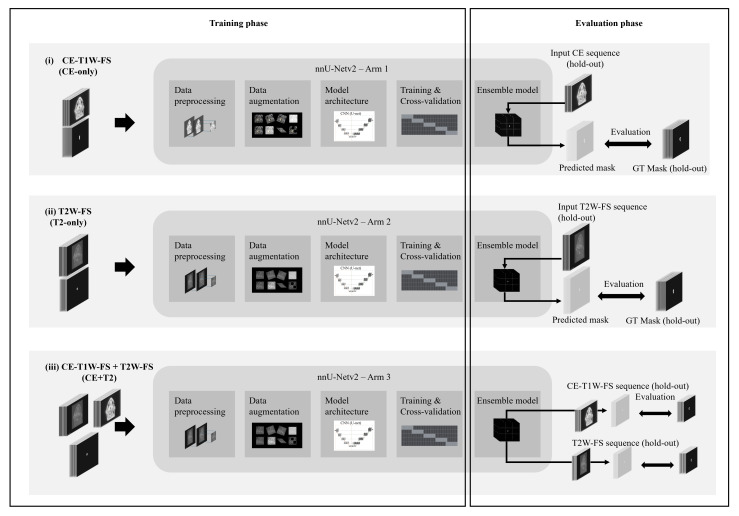
Per-outer-fold study design for sequence-specific training and evaluation: Independent arms, (**i**) CE-T1W-FS, (**ii**) T2W-FS, and (**iii**) CE-T1W-FS + T2W-FS, undergo the same pipeline (resampling/z-score/patching, spatial and intensity augmentations, nnU-Netv2 3D U-Net, five-fold CV with probability-averaged ensemble). For each outer fold, predictions are evaluated on paired hold-out scans against sequence-matched ground truths, enabling pairwise comparisons.

We trained and evaluated the models using three sequence-specific configurations (Figure 4): (i) CE-T1W-FS, using only contrast-enhanced sequences (CE-only), (ii) T2W-FS, using only non-contrast sequences (T2-only), and (iii) CE-T1W-FS + T2W-FS, using both sequences (CE + T2). The outer and inner partitions were kept consistent across the configurations to enable pairwise comparisons.

### 2.4. nnU-Net

We utilized the self-configuring pipelines from nnU-Netv2 (v2.6.2) [10] for pre-processing, augmentation, hyperparameter tuning and inference. We chose the 3D U-net to leverage the inter-slice information and training length of 150 epochs to account for our limited sample size. Sliding-window inference was set to a default value of 50% overlap. The hyperparameters and preprocessing settings selected by the nnU-Net model were accepted without any modifications. No additional post-processing was applied to avoid removing true multifocal GTVs. The entire training and evaluation code is made publicly available to facilitate reproducibility at https://github.com/rbramkumar/opscc_segmentation (accessed on 16 January 2026).

### 2.5. Evaluation Methodology

**Segmentation performance:** The Dice score was chosen as the primary evaluation metric since it accurately captures segmentation quality and facilitates comparisons with other works. The Surface-Dice@2mm, which measures the percentage of predicted boundary within 2 mm of the ground-truth boundary, was chosen as the secondary evaluation metric to assess clinical relevance. Other boundary-based agreement and volumetric metrics such as the HD95, ASSD, and GTV error (absolute and percentage errors) are reported. Bland–Altman analysis was used to quantitatively describe the bias (systematic over- or under-segmentation) and the limits of agreement (mean ± SD).

**Performance comparison:** To assess the difference in segmentation performance when different sequences are used, the paired dice scores were compared using the exact Wilcoxon signed-rank test (SciPy v1.15.3) along with Hodges–Lehmann estimator of paired differences (Walsh averages) and 95% bootstrap CIs. The paired comparisons were carried out to evaluate the difference in performance between (i) CE + T2 vs. T2-only and (ii) CE-only vs. T2-only. While other metrics were also analyzed in the same paired framework these results were interpreted only as supportive evidence and not treated as separate confirmatory tests.

**Qualitative evaluation:** We conducted a reader study where the segmentations were qualitatively rated by a radiologist with >10 years of clinical experience on a 4-point ordinal scale (0 = reject, 1 = acceptable with major edits, 2 = acceptable with minor edits, 3 = acceptable as-is). For each patient, the radiologist was shown both sequences, the ground truths, and predicted segmentations, but was blinded to which underlying model produced those segmentations. Segmentations were rejected if there were any errors unlikely to be corrected with minor or major edits within the clinical setting, such as substantial under- or over-segmentation or implausible anatomy. The paired ratings were then compared between (i) CE + T2 vs. T2-only and (ii) CE-only vs. T2-only. The Wilcoxon signed-rank test was used for the comparison of paired ratings and McNemar test (statsmodels v0.14.5) was used to compare dichotomized proportion of at least “acceptable with minor edits”.

### 2.6. Reporting Guideline

This study follows the recommendations outlined in the CLAIM (Checklist for Artificial Intelligence in Medical Imaging) [26] (Supplementary Table S7).

## 3. Results

### 3.1. Patient Characteristics

The final cohort included thirty-nine patients and the age of this cohort ranged from 38 to 84 years with a median age of 64 years (IQR 58.5–73) (Table 1). There were thirty-four males (87.2%) and 5 Females (12.8%). This cohort consisted of patients with a mix of small, medium, and large tumors whose GTVs ranged from 0.4 to 46.4 mL with a median GTV of 9.6 mL (IQR 3.4–19.1). There were 10 patients (25.6%) with tumor stage T1, 11 (28.2%) with T2, 14 (35.9%) with T3, and 4 (10.3%) with T4. Most patients had nodal involvement where 27 (69.2%) patients with N1, 7 (17.9%) with N2, and 1 (2.6%) with N3 stage, but 4 (10.3%) had no nodal involvement (N0). Tumors were in tonsils (71.8%), oropharyngeal wall (59%), and tongue/tongue base (38.5%). One patient had a bilateral tumor located on their tonsils.

This cohort was partitioned into eight outer folds (with 4–5 patients in the hold-out sets). The median age across the folds ranged from 58 to 72 years and each fold contained patients with a mix of tumor stages (T and N categories) as summarized in Supplementary Table S2.

### 3.2. Manual Tumor Segmentations

All outlined tumor regions were in the base of the tongue, tonsils, soft palate, and lateral and posterior oropharyngeal walls and had the appearance of asymmetrical soft tissue masses with irregular margins. Outlined masses were in stages I to IV and demonstrated a slightly hyperintense and isointense MR signal on T2FS with irregular contours. Eleven large tumors had a surrounding zone of slight perifocal edema. On post-contrast T1FS, tumors showed mild homogeneous and heterogeneous peripheral enhancement. However, some large tumors and metastatic lymph nodes had cystic changes and heterogeneous contrast enhancement.

### 3.3. nnU-Net

The network architecture selected by the nnU-Net pipeline was the same across all configurations and included a 3D U-Net consisting of six encoder–decoder stages, Instance normalization, and Leaky ReLU activations. Each training step used a batch of two 32 × 224 × 224 patches with median voxel spacing ranging from 0.391 to 0.449 mm along the *x* and *y* directions (axial plane) and of 4 mm along the *z* direction (superior–inferior). The training pipeline included extensive data augmentation steps such as geometric transforms, intensity adjustments, and noise additions. The complete nnU-Net settings are included in Supplementary Tables S2–S5. Within the inner CV loop, the mean segmentation performance (Dice) ranged from 0.30 to 0.60 for CE-T1W-FS and 0.40–0.67 for T2W-FS (Supplementary Table S6).

### 3.4. Quantitative Evaluation

#### 3.4.1. Segmentation Performance

The median dice score for the combined CE + T2 configuration was 0.63 (IQR 0.33–0.79) (Table 2). The median Dice scores for the individual sequences were 0.6 (IQR 0.36–0.76) for T2-only and 0.55 (IQR 0.27–0.81). Similarly, the median Surface-Dice@2mm was highest for the combined sequence (0.62) followed by CE-only (0.6) and T2W-FS (0.57). The median HD95 and ASSD for combined, CE-only, and T2-only were 11.39, 15, 11.3 mm, and 2.71, 2.98, and 2.98 mm, respectively (Supplementary Figure S1). Distance metrics were undefined for one case each in T2 and CE + T2 arms since the model missed the tumor entirely.

#### 3.4.2. Performance Comparison

For the primary metric (dice coefficient), paired Hodges–Lehmann comparisons showed no significant differences: CE + T2 vs. T2-only showed a slight directional advantage for CE + T2 (HL median paired difference (Δ) = −0.03; 95% CI −0.06 to 0; *p* = 0.11) and CE-only vs. T2-only showed no performance difference (HLΔ = 0.00; 95% CI, −0.06 to 0.11; 0.98) (Table 3).

For Surface-Dice@2mm, the comparison showed a statistically significant drop in performance from CE + T2 to T2-only configuration, with an HLΔ of −0.04 (95% CI, −0.09 to −0.01; *p* = 0.03). However, the drop in performance from the CE-only to the T2-only configuration was not statistically significant. The HLΔ was −0.01 (95% CI, −0.09 to 0.11; *p* = 0.85).

#### 3.4.3. Dependence on Tumor Size

**Figure 5:**
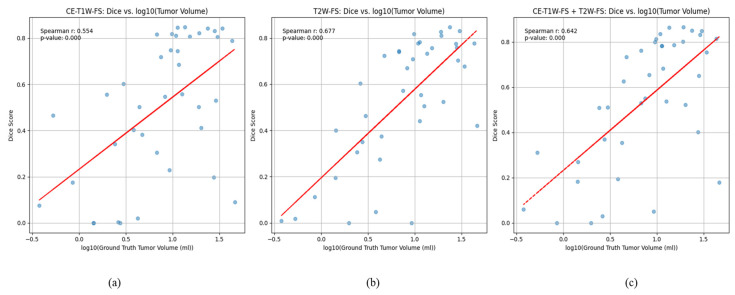
Relationship between tumor volume and segmentation performance: Scatterplots show Dice similarity coefficient versus log10-transformed ground-truth tumor volume (mL) for (**a**) CE-T1W-FS, (**b**) T2W-FS, and (**c**) CE-T1W-FS + T2W-FS configurations. Each point represents a single oropharyngeal squamous cell carcinoma case; red lines indicate linear fits for visualization.

The segmentation performance was better for larger tumors (Figure 5). The dice score correlated positively with the log-transformed GT Volume for all configurations, with moderate association for CE-only (Spearman 0.55, *p* < 0.001) and a slightly stronger association for T2-only (Spearman 0.68, *p* < 0.001).

#### 3.4.4. Subgroup Analysis: Small Tumors

The segmentation performance for small tumors (GTV ≤ 3 mL) is summarized in Table 4. The median Dice for T2-only, CE + T2, and CE-only were 0.25, 0.23, and 0.12, respectively. The median performance evaluated using Surface-Dice@2mm followed a similar pattern, with median values of 0.32, 0.31, and 0.22 for T2-only, CE + T2, and CE-only, respectively. The GTV agreement was highest for CE-only, followed by CE + T2 and T2-only, with ΔGTV (%) values of 5%, 39%, and 41%, respectively.

#### 3.4.5. Volumetric Agreement

**Figure 6:**
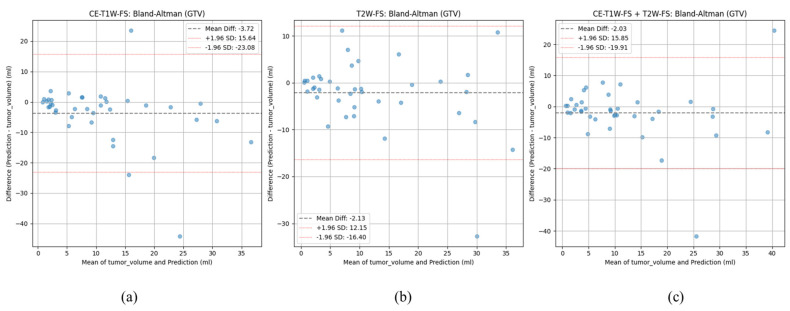
Bland–Altman plots for GTV (mL) for both training arms: (**a**) CE-T1W-FS, (**b**) T2W-FS, and (**c**) CE-T1W-FS + T2W-FS. The gray dotted line shows the mean difference, and the red dotted lines show the ±1.96 SD limits of agreement.

Volumetric agreement between the predicted GTV segmentation and the reference GTVs showed a slight tendency towards under-segmentation across all three sequence configurations (Table 2). The median absolute GTV volume differences were −1.31, −1.29, and −1.49 for the combination (CE + T2), T2-only, and CE-only, respectively. The corresponding percentage differences were −13%, −17% and −19% respectively. The lowest mean GTV difference was −2.03 ± 9.00 mL for the CE + T2 configuration, followed by −2.13 ± 7.19 mL for T2-only and −3.72 ± 9.75 mL for the CE-only configuration. The 95% limits of agreement showed slightly better volumetric stability for the T2-only sequence and were widest for the CE-only configuration (−23.08 to +15.64 mL), followed by the combined configuration (−19.91 to +15.85 mL) and T2 (−16.40 to +12.15 mL) (Figure 6).

### 3.5. Qualitative Evaluation

#### 3.5.1. Reader Study

For the 39 patients in this study cohort, qualitative review of the output segmentations was similar across CE-only, T2-only, and CE + T2. For CE-only, 6/39 (15.4%) segmentations were rated “acceptable as-is”, 9/39 (23.1%) as “acceptable with minor edits,” 15/39 (38.5%) as “acceptable with major edits”, and 9/39 (23.1%) as “reject.” For T2-only segmentations, the corresponding proportions were 6/39 (15.4%), 8/39 (20.5%), 19/39 (48.7%), and 6/39 (15.4%), respectively. Similarly, for CE + T2, the corresponding proportions were 4/39 (10.3%), 10/39 (25.6%), 16/39 (41%), and 9/39 (23.1%), respectively. For small tumors (GTV ≤ 3 mL), the rejection rates across the configurations were 5/10 (50%), 5/10 (50%) and 4/10 (40%) for CE-only, T2-only, and CE + T2 respectively.

Paired comparisons showed no significant differences between CE + T2 and T2 only (Wilcoxon *p* = 0.35) or between CE-only and T2-only (*p* = 0.75). The proportion rated at least “acceptable with minor edits” was identical for CE + T2 and T2-only (14/39 [35.9%] each; McNemar *p* = 1.00) and similar for CE-only vs. T2-only (15/39 [38.5%] vs. 14/39 [35.9%]; *p* = 1.00).

#### 3.5.2. Failure Analysis

**Figure 7:**
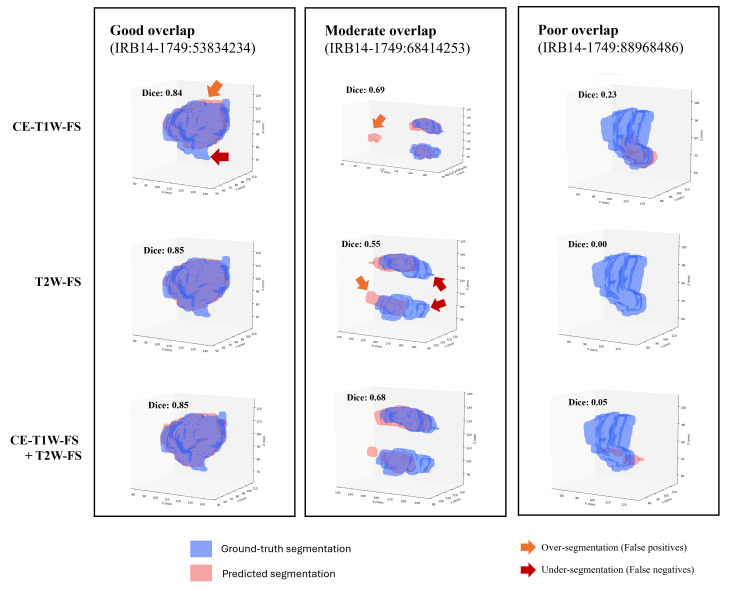
Representative examples of good, moderate, and poor overlap between ground-truth and predicted primary tumor segmentations. The patient IRB14-1749:68414253 (in center section) had a bilateral tumor present on their tonsils.

Representative images of cases selected with good and poor overlap are shown (Figure 7). In cases of good overlap, the predicted tumor contours were slightly smoother along the edges than the ground-truth contours. In cases of poor overlap, models either under-segmented (missed tumor voxels) or over-segmented (islands of false positives). 

**Figure 8:**
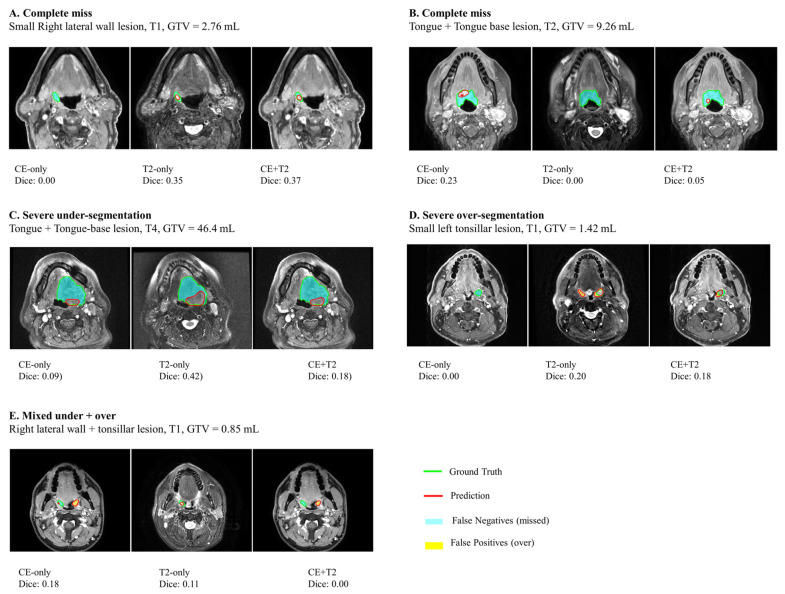
Representative segmentation failure modes across CE-only, T2-only, and combined CE + T2 models. (**A**,**B**) complete miss of the target lesion, (**C**) severe under-segmentation, (**D**) severe over-segmentation, and (**E**) mixed under- and over-segmentation within the same lesion. For each case, the axial slice with the largest ground-truth tumor extent is shown. Green contours denote the ground-truth segmentation, and red contours denote model predictions. Cyan overlays represent false-negative regions (missed tumor or under-segmentation), while yellow overlays represent false-positive regions (over-segmentation). Dice similarity coefficients are reported for each segmentation.

Rejected segmentations were classified based on the reason for rejection (complete miss, severe under-segmentation, over-segmentation/normal tissue inclusion, and mixed under- plus over-segmentation). Among the 24 rejected segmentations across all configurations, over-segmentation/normal tissue inclusion and mixed under and over were most common (7/24 each), followed by severe under-segmentation (5/24) and complete miss (5/24). Failures were more frequently observed in smaller tumors (<3 mL: 14/24) and early T-stage (T1: 14/24). The rejected segmentations were more frequently observed in tonsillar tumors (14/24), followed by tongue base (6/24) and lateral wall lesions (Table 5). Representative images of failure modes are illustrated in Figure 8.

## 4. Discussion

In this research, we evaluated the performance of an nnU-Net-based deep learning segmentation algorithm for automated segmentation of primary HPV-positive oropharyngeal squamous cell carcinoma (OPSCC) on contrast-enhanced T1-weighted (CE-T1W-FS), T2-weighted fat-suppressed (T2W-FS), and their combination. According to our results, all configurations showed moderate performance, highlighting the potential and limitations of automated segmentation approaches for small HPV-positive oropharyngeal tumors.

### 4.1. Comparison Between T1 FS Contrast-Enhanced and T2 FS Non-Contrast MRI Performance

Recent studies have developed radiomics models of autosegmentation OPSCC utilizing multiple MR sequences and multimodal approaches that incorporate MR, CT, and PET-CT [20]. Outeiral et al. [21] proposed a semi-automatic and fully automated segmentation approach of primary tumor segmentation on MRI sequences based on a 3D UNET architecture, which achieved a median dice of 0.74 and 0.64, respectively. Wahid et al. [22] proposed using anatomical segmentation (T1, T2) and functional (ADC, Ktrans, Ve) MRI sequences to improve segmentation boundaries. The study demonstrated the achievement of a mean Dice score of 0.71. Additionally, some studies have shown the potential to predict lymph node metastases [27,28], tumor HPV status [29], and predict outcomes of tumors, response to chemotherapy, and treatment strategies [30,31]. Corti A. et al. [32] offered a radiomic classifier of OPSCC gene expression and reflection of immune-infiltrating cells. This MR-based radiomic demonstrated promising results with an AUC of 0.92 [32]. Another recent study compared the performances of CT and MRI automatic segmentation of skull-base and neck tumors. The result showed that T1 with contrast-enhanced MRI had higher performance, with a median DSC of 0.58 (0–0.91) [33].

In this study, auto-segmentation using the non-contrast sequences performed comparably to contrast-enhanced and their combination, across multiple metrics, particularly Dice. Median Dice score in the current study is 0.55–0.62, broadly comparable but slightly lower than previous studies focused on OPSCC segmentation [21,22]. Several factors can explain these discrepancies. Wahid et al. [22] used a multiparametric approach including T1, T2, ADC, Ve, and Ktrans parameters, which potentially could improve tissue characteristics and segmentation accuracy. The current study compared the performances of T1 with contrast and T2 sequences. Our study included tumors with small and large sizes, and the median size was 0.4–46.4 mL, whereas in the previous study, it was 1.74–45.19 mL [22], which could potentially increase the accuracy of segmentations and performances in cases with small tumors. Additionally, sample size could impact Dice score performance. The sample size of another previous study was larger than our study, 39 patients, and included 171 patients, which better represents tumor variability and image heterogeneity.

While segmentation performance using both CE and T2 sequences was better than using only T2 sequences (HL ΔT2-combined −0.03; 95% CI −0.06, 0.00), these modest gains in Dice scores were not statistically significant, and narrow confidence intervals indicate that the advantage of adding contrast is likely small and unlikely to exceed ~0.06. There was no difference in segmentation performance between T1 contrast-enhanced and T2-only non-contrast sequences (HL ΔT2-CE 0.00; 95% CI −0.06, 0.11), but the confidence intervals are wider, which could indicate that there are slight gains and losses. These findings suggest that CE may not consistently demonstrate more precise segmentation, potentially due to differences in contrast uptake, image acquisition timing, tumor heterogeneity, and the appearance of HPV-positive tumors.

In this study, we also used Surface-Dice@2mm, which measures the proportion of the predicted contour surface within a 2 mm margin of the reference contour, as the secondary metric. We observed a statistically significant difference between CE + T2 and T2-only (HL ΔT2-combined = −0.04; *p* = 0.03), which suggests a slightly improved boundary alignment when CE is included. While this significance suggests a slightly better boundary agreement when CE is included, the magnitude of this improvement is modest, and more importantly, there were no concordant improvements in the primary Dice metric. We therefore interpret this significance only as an incremental boundary agreement. Additionally, the significant improvement in Surface-Dice@2mm with no significant improvement in Dice could suggest that the CE sequences provide important boundary localization information rather than bulk overlap. Future work can focus on carrying out targeted sub-group and qualitative analysis to test such hypotheses.

The quality and accuracy of segmentation significantly depend on tumor size. All three training configurations had higher Dice scores in segmentations of larger tumors and had moderate-to-strong correlations between tumor volume and performance. Small primary tumors, common in HPV OPSCC, remain a significant challenge for auto segmentation models, likely due to limited conspicuity, partial-volume effects, and increased sensitivity to slice thickness and through-plane anisotropy. The qualitative review demonstrated that most predictions required minor or major edits before being used in clinical practice. The segmentation performance of small tumors (GTV ≤ 3 mL) was relatively poor across all three configurations with median Dice scores (*n* = 10) ranging from 0.125 to 0.251 and had higher rates (40–50%) of rejection than that of all tumors (~15–23%) within the qualitative reader study. However, the GTV agreement (ΔGTV) within small tumors was higher in CE (5%) than in CE + T2 (31%) and T2-only (41%). Additionally, the qualitative review showed that the predicted tumor segmentations tended to have smoother boundaries than the ground-truth segmentations, which was a source of consistent under-segmentation. This can be expected since the nnU-Net algorithm was trained using the Cross-entropy and Dice loss functions, and therefore, the predicted segmentations tended to learn the volumetric/bulk overlap better than accurate tumor margins.

### 4.2. Clinical Significance. Practical Workflow Integration

Accurate segmentation of the primary tumor in oropharyngeal squamous cell carcinoma is essential for the planning of radiation treatment. Manual delineation remains time-consuming [34] and variable among clinicians. According to a recent study, AI-based autosegmentation significantly improved the time of delineation and demonstrated high clinical accuracy and reduced dosimetric impact. Integration of AI-based delineation tools in the routine practice in the field of radiotherapy promises improvements in efficiency [35] and interobserver variability [36].

Our study demonstrated that a fully automated nnU-Net-based segmentation can delineate tumor contours with moderate accuracy, particularly for larger tumors, and can generate preliminary contours that could potentially serve as a starting point for clinical review. Despite moderate performance across all sequences, T2-weighted and combined CE + T2 MRI sequences show significant potential to assist contouring workflows. Additionally, our results suggest that automated segmentation pipelines may be deployed effectively for non-contrast MRI. Models based on non-contrast sequences can particularly benefit patients with chronic renal disease or severe allergic reactions, limitations to access to contrast, and address cost-related concerns. In summary, the findings support the use of AI-assisted segmentation in HPV-positive OPSCC and lay the groundwork for the future development of deep learning models that may significantly reduce contouring time, improve consistency, and enhance the efficiency of radiotherapy planning.

### 4.3. Limitations

This study has several limitations that should be noted. The first limitation is the small sample size, which potentially reduces statistical power. Additionally, the study has limitations in generalizability, such as the sample from a single center, which contains a predominantly male cohort (87.2%), which could potentially limit external validity. Apart from the above, the narrow cohort from a single institution may not represent the broader heterogeneity of OPSCC presentations, which could be encountered in routine clinical practice. The reliance on a single institution’s MRI acquisition protocol may bias the model toward specific contrast characteristics and spatial resolution, which potentially reduces the performance of external data. Multi-center studies with diverse patient populations, including HPV-positive and HPV-negative patients and scanner types, could increase the variety of OPSCC presentations and increase generalizability. This study focused solely on MRI-based autosegmentation without using multimodal imaging such as CT and PET-CT. A combination of these data could potentially enhance segmentation robustness. Additionally, although experienced radiologists created manual segmentations, inter-observer variability remains a slight potential source of bias.

### 4.4. Future Directions

Future studies should focus on expanding the dataset with large, multi-institutional, multi-vendor MRI cohorts to improve model robustness and generalizability. Given the dependence of tumor segmentation accuracy on tumor size, higher-resolution imaging inputs, super-resolution reconstruction, voxel-level uncertainty modeling, and architectures designed to accurately capture fine anatomic structures are required.

Advanced learning approaches such as transformer-based models, hybrid CNN-transformer architectures, or multi-scale attention mechanisms could achieve higher performance in automatic delineation. Further research should be performed to analyze results using the Surface-Dice@2mm metric to better evaluate boundary agreement. To improve segmentation performance at boundaries, a combination of volumetric and boundary loss functions should be explored to study segmentation accuracy in irregular boundaries. Additionally, a multimodal combination of images using CT, PET-CT, and DWI MRI sequences could be potentially beneficial for overcoming limitations related to soft-tissue contrast and heterogeneity. Finally, future studies should be performed to assess the impact of segmentation on clinical practice, including time savings, interobserver consistency, and treatment plan outcomes.

## 5. Conclusions

In this study, nnU-Net demonstrated moderate performance for the auto-segmentation of primary HPV-positive OPSCC across CE-T1W-FS, T2W-FS, and combined CE + T2 MRI sequences. The combination of CE and T2W showed the highest overall and median Dice scores; however, improvements over single sequences were small and not statistically significant for the primary metrics. The quality of segmentations strongly depends on tumor size, with substantially better performance for large tumors and difficulty in segmenting small tumors. Volumetric agreement analysis showed an underestimation of GTV across all configurations, and qualitative radiologist review confirmed that most of the segmentation required minimal manual correction before clinical use. These results suggested that nnU-net provides a reliable baseline approach and has the potential to assist radiologists in delineation; however, current automated segmentation performance for HPV-positive OPSCC still requires supervision before clinical deployment. Additionally, non-contrast MRI images have a high potential to contribute reliably to the automated segmentation of OPSCC. This could reduce dependency on contrast agents, lower imaging costs, minimize scanning time, and improve accessibility in resource-limited settings. Finally, future work should be performed with larger, multi-institutional datasets, improved strategies for small-tumor detection, and advanced multimodal sequences to improve auto-segmentation performance.

## Figures and Tables

**Table 1:** Baseline clinical and tumor characteristics of the study cohort and outer cross-validation hold-out folds.

Feature	Category/Statistic	Overall Cohort Summary
# Patients	N	39
Age (years)	Mean ± SD	64.9 ± 10.5
	Median (IQR)	64.0 (58.5–73.0)
	Range	38.0–84.0
Sex	F	5 (12.8%)
	M	34 (87.2%)
GTV (mL)	Mean ± SD	12.8 ± 12.0
	Median (IQR)	9.6 (3.4–19.1)
	Range	0.4–46.4
Tumor Location	Oropharyngeal Wall	23 (59.0%)
	Tongue/Tongue base	15 (38.5%)
	Tonsil	28 (71.8%)
HPV status	HPV +	39 (100.0%)
Stage M	Mo	3 (7.7%)
	Mx	36 (92.3%)
Stage N	N0	4 (10.3%)
	N1	27 (69.2%)
	N2	7 (17.9%)
	N3	1 (2.6%)
Stage T	T1	10 (25.6%)
	T2	11 (28.2%)
	T3	14 (35.9%)
	T4	4 (10.3%)

**Table 2:** Out-of-fold segmentation performance.

Metric	CE-T1W-FS (CE-Only)		T2W-FS (T2-Only)		Combination (CE + T2)	
	Median [IQR]	Mean ± SD	Median [IQR]	Mean ± SD	Median [IQR]	Mean ± SD
Dice	0.55 [0.27,0.81]	0.5 ± 0.3	0.6 [0.36,0.76]	0.53 ± 0.27	0.63 [0.33,0.79]	0.54 ± 0.28
Surface-Dice@2mm	0.6 [0.32,0.79]	0.52 ± 0.29	0.57 [0.41,0.74]	0.53 ± 0.26	0.62 [0.43,0.81]	0.57 ± 0.27
HD95 (mm)	15 [5.2,29.56]	25.65 ± 33.8	11.3 [5.01,29.48]	26.34 ± 36.24	11.39 [5,31.34]	29.14 ± 38.48
ASSD (mm)	2.98 [1.28,8.69]	7.73 ± 12	2.98 [1.31,10.77]	7.64 ± 11.2	2.71 [1.15,9.36]	7.26 ± 9.96
GTV Abs difference (mL)	−1.49 [−5.39,0.52]	−3.72 ± 9.75	−1.29 [−4.07,0.65]	−2.13 ± 7.19	−1.31 [−3.17,0.91]	−2.03 ± 9
GTV (% difference)	−19 [−57,13]	22 ± 182	−17 [−41,33]	15 ± 137	−13 [−34,26]	28 ± 147

**Table 3:** Paired comparison results.

Metric	Comparison 1—CE + T2 vs. T2-Only			Comparison 2—CE-Only vs. T2-Only		
	# Patients (*N*)	HL Δ T2-Combination (95% CI)	*p*-Value	# Patients (*N*)	HL Δ T2-CE (95% CI)	*p*-Value
Dice	39	−0.03 (−0.06,0)	0.11	39	0 (−0.06,0.11)	0.98
Surface-Dice@2mm	39	−0.04 (−0.09,−0.01)	0.03	39	−0.01 (−0.09,0.11)	0.85
HD95 (mm)	38	0.29 (−2.76,2.37)	0.68	38	−0.46 (−3.83,1.9)	0.56
ASSD (mm)	38	0.31 (−0.84,2.22)	0.27	38	−0.02 (−1.88,3.08)	0.97
GTV difference (mL)	39	−0.42 (−1.29,0.42)	0.32	39	0.83 (−0.78,3.76)	0.41
GTV (% difference)	39	−3 (−13,8)	0.49	39	9 (−14,3)	0.39

**Table 4:** Sub-group analysis: Small tumor (<3 mL) segmentation performance (median [IQR]).

Metric	CE-T1W-FS (CE-Only)	T2W-FS (T2-Only)	Combination (CE + T2)
*N* (cases)	10	10	10
Dice	0.125 [0.001–0.434]	0.251 [0.041–0.389]	0.226 [0.038–0.355]
Surface-Dice@2mm	0.219 [0.009–0.579]	0.319 [0.117–0.501]	0.314 [0.110–0.469]
HD95 (mm)	28.1 [21.4–34.1]	28.0 [17.1–36.0]	32.1 [24.4–36.3]
ASSD (mm)	8.9 [4.5–14.9]	10.1 [4.5–13.2]	10.0 [8.4–11.8]
ΔGTV (mL)	0.08 [−0.89–0.73]	0.46 [−0.72–1.09]	0.38 [−0.61–2.20]
ΔGTV (%)	5 [−0.31–0.47]	41 [−0.23–0.75]	39 [−0.19–2.86]

**Table 5:** Failure mode characterization and stratification (rejected only) for all three configurations (CE-only, T2-only and CE + T2).

Characteristic	Complete Miss	Severe Under-Segmentation	Over-Segmentation/Normal Tissue Inclusion	Mixed Under and Over
Total Rejected *n* (%)	5 (20.8%)	5 (20.8%)	7 (29.2%)	7 (29.2%)
Tumor Size				
<3 mL	4	1	4	5
3–10 mL	1	1	3	2
>10 mL	0	3	0	0
Subsite				
Tonsil	2	2	4	6
Tongue base	2	3	1	0
Lateral wall	1	0	2	1
T-stage				
T1	4	1	4	5
T2	1	1	3	2
T3	0	1	0	0
T4	0	2	0	0

## Data Availability

The data presented in this study is available on request from the corresponding author.

## Supplementary Materials

The following supporting information can be downloaded at: https://www.mdpi.com/article/10.3390/diagnostics16050658/s1, Table S1. Scanner parameters; Table S2. Baseline clinical and tumor characteristics of the study cohort and outer cross-validation hold-out folds; Table S3. nnU-Net preprocessing characteristics by outer cross-validation fold for contrast-enhanced T1-weighted fat-suppressed (CE-T1W-FS) and T2-weighted fat-suppressed (T2W-FS) sequences; Table S4. nnUNet plan parameters and hyperparameters across dataset arms and outer folds; Table S5. nnUNet DA5 Trainer augmentation steps; Table S6. nnUNet Inner CV performance summary; Table S7. CLAIM Checklist; Figure S1. Box plots of segmentation performance metrics; and Code repository: https://github.com/rbramkumar/opscc_segmentation (accessed on 16 January 2026).

## Abbreviations

The following abbreviations are used in this manuscript:

GBCA	Gadolinium-based contrast agents
CNN	Convolutional Neural Networks
OPSCC	Oropharyngeal Squamous Cell Carcinoma
CE-T1W-FS	Contrast-Enhanced T1W Fat Suppressed
T2W-FS	T2W Fat Suppressed
HD95	Hausdorff Distance (95th Percentile)
ASSD	Average Symmetric Surface Distance
GTV	Gross Tumor Volume
HL	Hodges–Lehmann Estimator
