# Supplementary figures and images for: Deep Learning (nnU-Net)-Based Segmentation of Primary HPV-Positive OPSCC: Contrast-Enhanced T1-Weighted Fat-Suppressed Versus Non-Contrast-Enhanced T2-Weighted Fat-Suppressed MRI (Paired Single-Center Study)
Source: Diagnostics (Basel). 2026 Feb 25;16(5):658. doi: 10.3390/diagnostics16050658 (PMC12985114; doi:10.3390/diagnostics16050658)

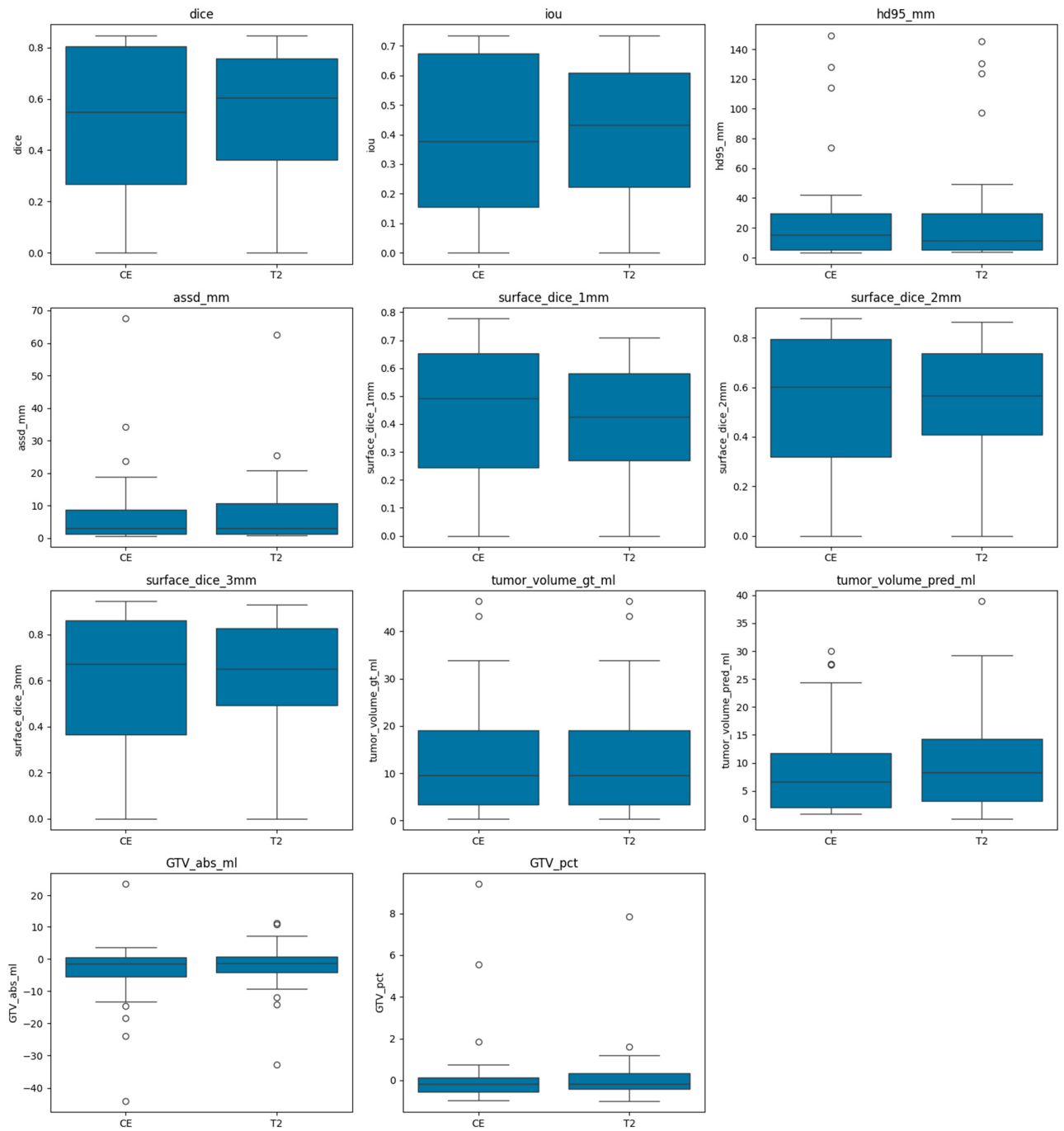

**Supplementary Figure S1.** Box plots of segmentation performance metrics

Supplement: Supplementary file 1 [file diagnostics-16-00658-s001.zip › Figure S1.pdf]
